# Physician Perspectives on the Potential Benefits and Risks of Applying Artificial Intelligence in Psychiatric Medicine: Qualitative Study

**DOI:** 10.2196/64414

**Published:** 2025-02-10

**Authors:** Austin M Stroud, Susan H Curtis, Isabel B Weir, Jeremiah J Stout, Barbara A Barry, William V Bobo, Arjun P Athreya, Richard R Sharp

**Affiliations:** 1 Biomedical Ethics Program Mayo Clinic Rochester, MN United States; 2 Alix School of Medicine Mayo Clinic Rochester, MN United States; 3 Robert D. and Patricia E. Kern Center for the Science of Health Care Delivery Mayo Clinic Rochester, MN United States; 4 Department of Behavioral Sciences & Social Medicine College of Medicine Florida State University Tallahassee, FL United States; 5 Department of Molecular Pharmacology and Experimental Therapeutics Mayo Clinic Rochester, MN United States

**Keywords:** artificial intelligence, machine learning, digital health, mental health, psychiatry, depression, interviews, family medicine, physicians, qualitative, providers, attitudes, opinions, perspectives, ethics

## Abstract

**Background:**

As artificial intelligence (AI) tools are integrated more widely in psychiatric medicine, it is important to consider the impact these tools will have on clinical practice.

**Objective:**

This study aimed to characterize physician perspectives on the potential impact AI tools will have in psychiatric medicine.

**Methods:**

We interviewed 42 physicians (21 psychiatrists and 21 family medicine practitioners). These interviews used detailed clinical case scenarios involving the use of AI technologies in the evaluation, diagnosis, and treatment of psychiatric conditions. Interviews were transcribed and subsequently analyzed using qualitative analysis methods.

**Results:**

Physicians highlighted multiple potential benefits of AI tools, including potential support for optimizing pharmaceutical efficacy, reducing administrative burden, aiding shared decision-making, and increasing access to health services, and were optimistic about the long-term impact of these technologies. This optimism was tempered by concerns about potential near-term risks to both patients and themselves including misguiding clinical judgment, increasing clinical burden, introducing patient harms, and creating legal liability.

**Conclusions:**

Our results highlight the importance of considering specialist perspectives when deploying AI tools in psychiatric medicine.

## Introduction

The use of artificial intelligence (AI)—“a machine-based system that can, for a given set of human-defined objectives, make predictions, recommendations, or decisions influencing real or virtual environments” [1]—in psychiatric medicine has been of much interest to clinicians, researchers, and developers, with potential to support areas such as pharmacological treatment [2] and psychotherapy [3]. Particularly, machine learning—“a set of techniques that can be used to train AI algorithms to improve performance at a task based on data” [1]—methods combining clinical, sociodemographic, and biomarker data (eg, pharmacogenomics) to predict treatments, prognoses, and diagnoses have been proposed for psychiatric care [4,5]. Similar methods have been used to develop screening tools that identify psychiatric disorders such as major depressive disorder (MDD) and generalized anxiety disorder, while AI tools using natural language processing aim to provide access to therapy and mental health guidance [6-8].

Despite the potential of these AI tools, there is varying evidence supporting their adoption in clinical practice. For instance, digital mental health applications have proliferated in consumer marketplaces and yet have mixed evidence supporting [9] and disputing [10] their therapeutic efficacy. In addition, AI-enabled clinical prediction tools for precision psychiatry, while having improved over the last decade, face challenges in clinical translation related to bias and overfitting, generalizability, and clinical use [11]. In addition to these evidentiary considerations, there are ethical concerns related to patient safety, transparency, data privacy, bias, and responsibility that present additional implementation challenges [12,13]. While these tools are not yet standard of care, they are gaining acceptance and likely to impact psychiatric medicine in a variety of ways in the future [14,15].

It is unclear how physicians treating patients with psychiatric conditions will respond to these new AI tools. Health systems are experiencing resource constraints amid great demand for psychiatric services [16,17], with AI increasingly being viewed as an opportunity for providing support [18,19]. These technologies may change practices by altering conventional workflows, training, and decision-making for physicians [20]. As with applications of AI in other clinical specialties, accounting for physician perspectives in psychiatric medicine will be critical for addressing their specific needs as practitioners and primary end users of these tools. Key considerations for acceptability and integration of AI may involve physicians’ assessments of the benefits and risks that will be introduced to both themselves and their patients [21,22].

Prior studies have examined physician attitudes broadly toward AI often focusing on general acceptability [23-26]. In addition, some psychiatry-focused research has begun to characterize physician use of AI-enabled clinical support tools or clinical decision support systems for depression [27,28], as well as physician perspectives on AI in adolescent care [29] and generative AI [30]. In this paper, we report results from a qualitative research study that sought to characterize physician perspectives on applications of AI in psychiatric medicine. Drawing on several emerging real-world applications of AI in psychiatry, our study offers insight into perceived benefits and challenges that specialists are likely to consider in the adoption of AI tools. This study aims to advance the understanding of AI tools in psychiatric care by leveraging in-depth interviews to uncover the nuanced ways frontline physicians perceive and anticipate impacts of AI in their clinical practice.

## Methods

### Participant Recruitment

Physicians were recruited from a single health care system in the Upper Midwest of the United States. We identified eligible participants by searching the health system’s enterprise health record database for physicians in family medicine or psychiatry who met the following criteria: (1) prescribed selective serotonin reuptake inhibitors for depressive disorders and (2) diagnosed patients using *ICD-10* (*International Statistical Classification of Diseases, Tenth Revision*) codes relevant to MDD (F32, F33, F34.1, N94.3, F32.8, and F32.9). MDD was relevant to several of our case scenarios and served as a suitable entry point for discussing broader psychiatric conditions and accompanying AI tools with physicians. Family medicine practitioners were included in addition to psychiatrists given the high incidence of psychiatric conditions that are diagnosed and treated in the specialty [31,32]. Participants must have made a minimum of 1 prescription and 15 or more diagnoses during their tenure. This ensured that participants had some experience with the clinical subject matter discussed in the interviews. Physicians returned in the search were randomized and recruited via email invitation. Interviews were conducted between February 1, 2022, and April 28, 2022.

### Ethical Considerations

This study was approved by the Mayo Clinic institutional review board (protocol 21-006191). All participants consented orally to participation in the study and to have their interviews recorded. All data reported have been deidentified. Participants voluntarily enrolled in the study. No compensation was provided.

### Data Collection

#### Case Scenarios

Four hypothetical clinical case scenarios were provided that described various AI tools in psychiatric medicine settings [33]. The cases included depictions of AI tools that (1) assisted a physician in prescribing medications based on patient pharmacogenomic information, (2) provided adjunct cognitive behavioral therapy to a patient via a chatbot interface, (3) assisted a physician with providing a differential diagnosis and associated disease risk scores, and (4) identified patients at risk for suicide based on population health characteristics. Initial cases were constructed based on a review of commercially available tools and academic literature. The depicted technologies were chosen to highlight varying health conditions and use cases for psychiatric AI tools. In accordance with methodological best practices, cases were subsequently revised and finalized in consultation with subject matter experts in psychiatry to confirm their relevance to clinical practice [34]. Cases were also evaluated during interviews with direct questions posed to participants: “Did the cases presented to you seem like something you might encounter in your practice?” “Did any of the cases seem implausible for any reason?”

#### Interviews

We conducted in-depth interviews following a case-based approach [35,36]. All interviews were conducted virtually over Zoom (Zoom Video Communications) with 2 study team members present. One primary interviewer (SHC) led the interviews, and 1 of 2 secondary interviewers (AMS and JJS) took notes and asked follow-up questions. The interviews began with questions that explored participant understanding, familiarity, and experience with health care AI such as “Can you briefly tell me about your familiarity with the idea of using AI tools in healthcare?” A formal definition of AI was not provided to participants. Cases were introduced after these general questions along with several structured questions gauging physician perceptions of the AI tool such as “What do you think the benefits of this tool could be?” and “What potential risks or concerns does this tool pose?” Due to time constraints, not all cases were discussed in each interview. Interviewers selected cases to ensure that each one was presented in interviews throughout the study. Additional questions focused on physicians’ assessments of AI and included questions such as “What risks and benefits do you think AI could bring to your practice?” This approach allowed us to explore specialist attitudes toward AI in psychiatric medicine and in health care generally. Questions and probes were designed to be open-ended to encourage exploration of participant viewpoints. Throughout data collection, the study team met regularly to refine interview questions and address gaps in the data, helping to support thorough data collection.

Interviews were audio-recorded and externally transcribed by a professional transcription firm. Transcripts were subsequently deidentified and edited by the study team for any typographical errors or other transcription inaccuracies by reviewing the transcript alongside the original audio.

### Data Analysis

Interview data were analyzed following an inductive approach. A field note was created summarizing general themes after each interview [37]. Upon completing the set of 42 interviews, a synthetic field note was created to summarize emerging themes across interviews. A preliminary codebook was then created from the individual field notes, synthetic field note, and interview guide. This preliminary codebook was applied to a subset of the transcripts that varied in length, interview number, cases used, and physician specialty. The performance of the preliminary codebook was discussed by the study team including reflective assessments of intercoder agreement as well as the codebook’s coverage, consistency, and clarity. After these study team discussions, the codebook was revised into a final version that was applied to the set interviews. Throughout coding, the final codebook was iteratively revised and applied as themes emerged in the dataset [38]. The coding team consisted of 1 primary coder who analyzed the full set of transcripts (IBW) and 2 secondary coders who each coded a subset of transcripts (AMS and SHC). Transcripts were coded independently and in duplicate using NVivo (release 1.7.2; Lumivero, LLC). After independently coding, primary and secondary coders subsequently met to reach consensus [39]. Once interview data were fully coded, the study team discussed and refined several emerging themes related to potential benefits and risks of AI tools.

## Results

### Overview

We contacted 143 physicians to participate in an interview. Forty-two physicians accepted and enrolled in the study (21 practicing in psychiatry and 21 in family medicine). Our total participation rate was 29.37% (42/143 physicians invited). Major themes from their perspectives on benefits and risks of AI in psychiatric medicine along with their overall assessments are described in the following sections. While physicians often responded in the context of the presented cases, they also speculated on other potential AI tools and how they might affect their clinical practice.

### Benefits

Physicians identified multiple potential benefits to the adoption of AI tools in psychiatric medicine. These were interpreted as benefits related to clinical support, administrative burden, patient needs, and health access.

#### Physicians Believed AI Could Provide Valuable Clinical Support

Participants noted that AI may be helpful in supporting clinical decision-making. Physicians explained the potential for AI to aid diagnoses and treatment and discussed how it could add another layer of clinical assessment. In the context of psychiatric diagnoses, physicians suggested that AI may support them where their insights be limited. “AI can help classify people’s major depression, but maybe there’s subcategories of major depression that I’m not smart enough to discern [...]” (Interview 24, Psychiatry). Participants also described the potential for AI to mitigate errors such as missed symptoms or provide a set of default actions or recommendations that may be able to identify issues that could go undetected.

*I think it [AI diagnosis tool] would be brilliantly helpful in reminding us that, for instance, [...] cannabis use looks like ADD, so with the racing thoughts and these sorts of things. I think to remind us all the time, like, in a casual conversation I didn’t ask her that.*

Interview 32, Family Medicine

Physicians felt that AI could be potentially helpful in synthesizing information on patients to guide treatment. Participants also discussed personal limitations to their clinical knowledge and noted that AI could offer diagnostic and treatment insights based on data that exceed their range of clinical experiences. “That [AI diagnostic tool] might help to guide what treatments I recommend, drugs or psychotherapy or behavioral activation or other factors” (Interview 17, Psychiatry). Participants noted that AI could offer opportunities for guidance in situations where physicians are uncertain about treatment plans.

*It [AI diagnosis tool] could augment the clinical assessment because our attention, our working memory, our problem-solving capacity is limited by the built-in, beautiful things and inefficiencies of the human mind [...] It helps us add another layer of empirically validated reasoning that will help us overcome the shortsightedness that we inherently have.*

Interview 37, Psychiatry

#### Physicians Believed AI Could Alleviate Administrative Burden

Participants described how AI could reduce their administrative burden by offloading tasks such as clinical documentation and information synthesis. Many physicians expressed hope that using AI would decrease their overall administrative workload and allow them to spend more time on direct patient care.

*If there’s a way for AI to help with documentation and administrative burden, and do some of that for you 'cause I think that’s where a lot of burnout in practice is. It’s not having to see a bunch of patients. It’s having to do all the paperwork that goes with it.*

Interview 02, Family Medicine

Physicians voiced the potential for AI to support their clinical workflows such as improving the efficiency of data access. AI tools were also thought to be useful for information gathering and helping to retrieve more quality data that could be used for clinical decisions.

*I think that [AI diagnosis tool] would be useful as well, simply, because a lot of those different conditions that you had mentioned have very specific DSM criteria to meet that diagnosis. It’s hard for any one person to have all of that in their head at one time [...].*

Interview 02, Family Medicine

Some physicians highlighted that there would be considerable use of such tools in particularly complex clinical situations.

*I’m going to look through the system [AI diagnosis tool] and say, hey, what does the system say about this? If I encounter a case that it’s so tough for me that I can’t figure anything out, then maybe I’ll wanna do that because then that’s a more efficient use of my time.*

Interview 19, Psychiatry

Some participants even suggested that aspects of their practice could be automated by allowing AI to handle certain tasks.

*I think it [AI] can, hopefully, cut out doctors when they’re not needed but also bring to light important pieces of information to doctors when it would be helpful. It can also help guide treatment and get people the right care at the right time [...] rather than just fatiguing doctors with all the alarms and warnings all the time that I just quickly click through to get them out of my face, it might be helpful to just automate a lot of these things.*

Interview 06, Family Medicine

#### Physicians Believed AI Could Support Patient Needs

Physicians noted the potential for AI to provide accurate and early diagnosis of psychiatric conditions and shape initial treatment recommendations. “I think that [AI pharmacogenomics tool] would be an excellent way to be able to help people choose what, hopefully, is the most successful medication right off the bat” (Interview 11, Family Medicine). Beyond the individual use cases we discussed, physicians also extrapolated broader benefits of other AI technologies being applied in psychiatric medicine.

*I know thinking of psychiatric care and depression care, if there was an AI tool that helped pick more accurately the most successful antidepressant or something like that, that is a challenge that we deal with every day, and something that could have immediate results.*

Interview 04, Family Medicine

Participants commented on the potential for AI tools to support shared decision-making conversations with patients. This was often explained by noting that the additional evidence provided by AI could be empowering for clinical recommendations as both patients and clinicians will have another data point to evaluate when considering a decision.

*Sometimes it helps me to show patients, too. I can say, “Hey, look right here. The computer says it’s worried about you having this more serious problem. We need to do some more testing.” It can help persuade some of those people that it’s not just me telling them that I have this idea and “I just wanna order more tests,” or “I think we should be on a different medicine.” If I have sometimes some of that information on the computer, it’s useful.*

Interview 11, Family Medicine

Participants highlighted how AI might reduce common patient barriers to seeking treatment for psychiatric concerns, particularly in cases where behavioral therapy would be appropriate. They noted that patients might consider AI preferable to conventional human-directed therapy due to additional barriers such as stigma, cost, or inconvenience.

*Going to therapy or even seeing a psychiatrist for many people is super scary. It makes people feel vulnerable and metaphorically stripped naked, and if they could interface with an app [...] it might be a way to get someone care earlier and more effectively than not having an interaction at all because of fear of coming in, or having to take time off of work, or any number of reasons that are barriers to people seeking mental healthcare.*

Interview 17, Psychiatry

#### Physicians Believed AI Could Expand Health System Access

Physicians discussed factors such as declining availability of specialty providers, increasing demand from patients, and lingering effects of the COVID-19 pandemic as limitations to current behavioral health systems.

*There’s a lot of backlog and waiting, especially with the pandemic, to have that [psychiatric] care, so it’s a big barrier. Access is a big barrier. I think, in this specific instance, using AI [chatbot tool] that comes from a provider might be helpful to, again, get people care.*

Interview 35, Family Medicine

Participants noted opportunities for AI to expand access to psychiatric support by providing another way for patients to access services that they would otherwise be unable reach through conventional means. “The ability to do it [therapy] on your phone on your own time at home with something like a chatbot does seem super-convenient [...] especially in rural communities where there’s not great access” (Interview 04, Family Medicine). In addition to supporting areas where resources might be limited, participants noted that AI tools might aid patients who are unable to connect with a clinician in the near-term.

*We talked a minute ago about how unavailable therapists are. I hear that all the time that even in a town as provider-rich as [City] is, people can’t get in. If there is an electronic application [chatbot tool] that’s proven to help, probably it’s a good thing.*

Interview 10, Family Medicine

Participants voiced the potential for AI to extend the capabilities of general practice physicians, who are increasingly involved in providing psychiatric care but who may have limited formal training in psychiatric medicine. Physicians also reflected on the potential impact AI could have on health systems, noting that AI support for general practitioners could aid their capacity to assist patients with complex psychiatric needs.

*Yeah, because they’re [primary care physicians are] dealing with a lotta psychiatry. They are very under trained. [...] They’re like, “What? I have no idea. I had one month of training in med school. What am I supposed to do with this?” If there is a way to attack that with AI, that would be really helpful.*

Interview 28, Psychiatry

In addition to supporting general practice physicians, participants also commented on how AI tools might allow psychiatric specialists to handle more complex care needs.

*Really, anything that extends the ability of psychiatry to take care of the more complex patients and not deal with the day-to-day routine stuff would be wonderful. I mean, we’re looking at a massive die-off of psychiatrists in the near future, and there’s not gonna be enough of us around [...].*

Interview 25, Psychiatry

### Risks

Physicians also identified discrete risks with the adoption of AI tools. These perceived risks related to how AI tools might be unreliable, could increase burden, result in patient harm, or create medicolegal issues.

#### Physicians Were Concerned AI Could Be Unreliable

Physicians expressed some apprehension toward AI due to concerns that these systems might produce unreliable recommendations. They discussed the importance of having strong underlying research and data to support AI that informs clinical decision-making and noted that improperly developed tools or skewed data could lead physicians to make poorly informed decisions.

*If we don’t have a really robust analysis of the things that determine health for that patient informing the decision making, then I think we would run risk of making inappropriate decisions or having that data be harmful perhaps.*

Interview 16, Family Medicine

Participants mentioned concerns over the capabilities of AI compared with human practitioners, often considering whether these technologies would be acceptable for guiding patient care. Chatbots, for instance, were sometimes viewed as being incapable of capturing aspects of therapeutic relationships.

*With psychotherapy, in particular, a big part of the concept is having an experience with another person who’s helping you that can then generalize to other relationships that you have. While I think some concepts can come out of dialogue, so to speak, with a chatbot, I am not convinced that being able to trust a human being can be accomplished by being able to trust an app [...].*

Interview 17, Psychiatry

Some physicians were skeptical that AI could produce rigorous decisional support that would be comparable with advice generated by a physician.

*My reservations, I guess, have to do with uncertainty about the flexibility and the reliability of those algorithms and whether a system would recognize as well as a human practitioner when that algorithm may not be serving the patient best, and that some other approach may be necessary.*

Interview 24, Psychiatry

Participants also noted problems that could arise from physicians being overly reliant on AI in the future. They explained the potential for physicians, particularly those with less experience, to become dependent on AI and fail to properly develop their clinical skills.

*[...] if you roll this [AI diagnosis tool] out in a residency clinic and the clinicians at that time get used to using an AI system and then going out to a rural practice where maybe that AI system is not available [...] maybe they don’t know exactly how to come up with that diagnosis or everything because they’re relying on artificial intelligence versus rote memory.*

Interview 41, Family Medicine

They noted that this dependence could cause additional problems if AI systems turn out to be faulty. Furthermore, participants expressed concerns about physicians consistently deferring to AI recommendations without considering additional patient factors beyond what is required by a model.

*I think risks would be if you utilize—you relied on it heavily, black and white, and you didn’t consider the other aspects of the patient that aren’t going into the algorithms.*

Interview 05, Family Medicine

#### Physicians Were Concerned AI Could Increase Burden

Physicians expressed concerns that AI could be a source for additional administrative burden by adding to their clinical workflows. Some participants noted that AI could potentially overwhelm physicians with data, making it difficult to interpret recommendations. In addition, physicians were also concerned about additional data collection and entry requirements in support of AI tools noting that these requirements could be disruptive to their clinical encounters.

*If I need to click 60 click-boxes as far as symptomatology to get the AI tool to tell me this sounds more like anxiety than bipolar or depression, that’s just a clerical burden that’s hard to overcome in primary care, where time is short in most visits.*

Interview 04, Family Medicine

Participants also discussed how AI could ultimately lead to an increase in their clinical workload. Physicians noted that additional training might be necessary to leverage AI systems, and that general practitioners might require additional psychiatric training to provide appropriate follow-up. Some physicians discussed the likelihood of an increase in the number of referrals to specialists because of new AI-enabled screening tools.

*The irony is that a tool that we think may be implemented to leverage service may wind up increasing our workload instead of relieving us of some workload. Patients are referred to us [...] and there’s nothing really abnormal. That doesn’t mean the screening tool is bad or that it shouldn’t be used, but there will be some false positives, I imagine, in that process.*

Interview 24, Psychiatry

Physicians also discussed the potential for AI tools to increase their burden by providing additional information but without accompanying resources to intervene. Participants commented that situations like these ultimately place a greater burden on physicians as they are provided insights but are unable to act and noted that AI insights alone are not enough to provide a benefit.

*You’re telling me you’re [suicide risk prediction tool] gonna identify this and you’re gonna give me the problem, give me the solution. Tell me what intervention’s gonna help prevent this suicide. Give me the resources available. If you say, “See a counselor and do this” and I don’t have a counselor, then it doesn’t do me any good. We have to really have the identified intervention named, the process and the resources for it.*

Interview 15, Family Medicine

#### Physicians Were Concerned AI Could Result in Patient Harm

Participants raised concerns that AI could result in clinical harms to their patients. Physicians were concerned about potential errors associated with poorly designed or unvalidated AI systems. Participants were also concerned about whether AI tools would be designed to be inclusive of their patient population.

*[...] we assume a lot of these tools are going in blind, and there are no biases, but I would be concerned about, is my algorithm racist, for instance, or other way is it disadvantaging—putting certain groups at a disadvantage?*

Interview 22, Psychiatry

In addition to clinical harm, physicians identified other risks including security and cost concerns. Participants considered patient data privacy, especially in circumstances where models leverage sensitive information related to psychiatric health concerns. “For example, that [chatbot] app [...] can get hacked, so those transcripts [are] basically getting breached” (Interview 09, Family Medicine). Physicians also identified the potential for cost increases to patients if AI-supported treatment recommendations included higher cost prescriptions or services than what might otherwise be assessed by a physician.

*[...] if it’s [AI] an added cost to the healthcare system, either directly or indirectly, whether it’s a waste of my time, and that time is a cost. If it’s a cost that’s gonna be incurred somewhere down the line in a patient’s billing, because we have to have this tool integrated into our system, I don’t think it would probably be worth the help there. *

Interview 08, Family Medicine

This was often raised as a concern in the pharmacogenomics case, where physicians considered varied costs based on the types of medications being recommended by the AI tool.

*[...] let’s say that the AI [pharmacogenomics] tool suggested a non-generic medication. Now, the patient is going to have a higher cost based upon that, and I guess we don’t know enough from the history, although you might’ve said that this is the first time the patient has ever had a depression.*

Interview 17, Psychiatry

#### Physicians Were Concerned AI Could Create Medicolegal Issues

Participants expressed concerns about liability in relation to AI-supported clinical recommendations. As expressed by one physician, “how do we defend that in court if something goes wrong?” (Interview 25, Psychiatry). Physicians had various perspectives on whether a flawed AI recommendation or system error would result in personal liability to them, their institution, or the AI developer.

*I think, ultimately, it’s still going to be the doctor. Because even if it’s an AI system, you know, the doctor is still supposed to be overseeing it [...] if the psychiatrist sits back and says, “I’m gonna trust the machine to do this,” and the patient kills themselves. It’s still the psychiatrist’s fault, you know.*

Interview 27, Psychiatry

Physicians noted potential for added complexity to their clinical decision-making given that there might be times where they disagree with AI-enabled recommendations. They discussed how in the treatment of psychiatric conditions there is often a need for additional assessment to verify indications from screening tools. “I think AI guided decisions will be wrong sometime. When that happens, who’s at fault?” (Interview 24, Psychiatry). Participants were wary of legal risks they would be exposed to when their clinical assessments and actions fail to align with AI recommendations.

*What if it [suicide risk prediction tool] flags and you talk to the patient and you get a sense by talking to them that they’re not acutely suicidal, but then they go off and kill themselves, right? Now my artificial intelligence identified this as a concern and does it show some type of liability.*

Interview 41, Family Medicine

### Overall Assessments

While participants shared both positive and negative perspectives on the potential impacts of AI tools, they generally saw these tools as promising in the care and treatment of patients with psychiatric conditions. Physicians were cautiously optimistic that future versions of health care AI could enhance the quality of their work and improve patient care.

*I think that I embrace it [AI]. I think it’s going to make us better psychiatrists. I think it’s gonna give us better quality information [...] I think it’s going to make us better therapists too. I hope that it’s gonna get better and better and better [...].*

Interview 26, Psychiatry

*I see down the line I’m cautiously optimistic about the use of it really helping to improve the efficiency and lower costs because we’re on an unsustainable growth curve of costs in health care right now.*

Interview 06, Family Medicine

Despite this general optimism, participants expressed concern about premature deployment of unproven AI tools in psychiatric medicine.

*I think I’m in agreement with the idea of developing these tools, but I don’t think the state of science is at the level that I would be supportive of them being used in a field of psychiatry.*

Interview 14, Psychiatry

*I feel like that time has not yet come, based on my experience with AI. Even the most intelligent computer I’ve seen [...] doesn’t feel natural [...] I feel like in the front end, I think, AI has a long way to go.*

Interview 21, Family Medicine

## Discussion

### Principal Findings

Our study contributes to the existing discussion of AI in health care with added nuance for applications in psychiatric medicine. Our results suggest that physicians are generally optimistic about the potential benefits of AI, but their optimism is tempered by concerns about potential risks to themselves and their patients. Some of the perceived benefits and risks identified in our study track with previous examinations of physician attitudes toward AI in health care broadly and prior survey research [40,41], suggesting that there may be a set of physician considerations, irrespective of the specialty using AI tools [42].

Our findings also support various theoretical models for technology acceptance in health care. Contributions such as the Technology Acceptance Model (and its iterations) provide a framework for examining user perspectives and intentions to adopt technologies and have been considered extensively in health care [43,44]. While the goal of this study was not to suggest a new or adapted model for AI tools, we highlight physician perspectives that may inform model constructs when applied to psychiatric medicine. For instance, our study identified that many physicians were cognizant of significant resource shortages and unmet patient needs for psychiatric care, often suggesting that AI could support these gaps. The perceived usefulness of AI tools in psychiatric medicine in the near-term might mediate these circumstances and potentially influence physician willingness to adopt such technologies. Other perceived benefits and risks identified by our study help situate the landscape of external variables that may facilitate or hinder the adoption of AI tools.

In addition to supporting theoretical models, our research advances previous work aimed at characterizing physician perspectives toward AI in psychiatric medicine. Survey research on general AI applications [40] and on generative AI [30] in psychiatry has contributed high-level considerations for physician attitudes. Our findings provide nuance and depth to some of these prior considerations. For instance, while health care access considerations have been identified in prior work, our findings detail exactly how physicians anticipate greater access will be achieved (ie, reducing stigma, enhancing convenience, and augmenting generalists). Furthermore, our results provide an understanding of potential use cases of AI tools in psychiatry from the perspective of specialists, enhancing broader understanding of how these tools might be supportive or harmful in practice. These considerations also advance previous work with psychiatrists and clinical decision support systems [41] by characterizing family medicine practitioner and psychiatrist perspectives on specific perceived risks in relation to AI tool acceptance.

One consideration for the degree of optimism toward AI tools is the backdrop of existing resource strain faced by behavioral health systems and practitioners [16]. While there may be potential for AI to shore up resource limitations in psychiatric medicine, there are still lingering questions about the fidelity of these tools and their net benefit to clinical practices [10,45]. Furthermore, there is added concern that a focus on AI to address issues in psychiatric medicine may obfuscate more systemic problems that are impacting the field. Our results show that physicians are still mindful of these systemic issues limiting psychiatric medicine and that these issues can remain even with adoption of AI.

Physicians identified areas where AI had the potential to both help and hurt. For instance, while many physicians saw a benefit to AI alleviating burden in their workflows, there was also consideration for these systems to create additional burden. This suggests that benefits and risks from adopting AI may not be uniformly experienced and physicians may simultaneously be both hopeful and cautious about the integration of AI in their practice. Developers and adopters of AI in psychiatric medicine should be cognizant of these simultaneously advantageous and disadvantageous circumstances along with potential unintended consequences [12]. It is important to consider that many behavioral health AI technologies are still in relatively early stages of technological maturity and that perspectives may evolve as AI tools are deployed in clinical practice [46], especially with the advancement of generative AI tools [30].

As health care organizations look to implement potential AI technologies, physicians will be key drivers of the success or failure for many of these tools [22]. Our research supports prioritizing physician involvement in AI development and adoption, where neglecting the perspectives of physicians can be a path toward failure [21]. Family medicine practitioners and psychiatrists will play an important role in managing the deployment of these AI tools, as they currently manage a wide variety of psychiatric health concerns.

### Limitations

The results and conclusions of this study must be viewed with limitations in mind. All participants were recruited from the same institution; thus, our results may not be reflective of physicians in different organizational and practice settings. Furthermore, we did not collect detailed participant demographic information outside of their specialty, diagnosis counts, and prescription counts, which were used for recruitment purposes. While this information may help characterize our participant sample, we did not intend to make conclusions or generalizations based on a traditional reporting of demographics (ie, participant age, sex, race or ethnicity, etc) and were primarily concerned with physician specialty due to the differences in scope of practice [47]. In addition, by including both family medicine practitioners and psychiatrists, our findings might be influenced by experiential differences of these groups and greater variability in responses. As a qualitative study, our methods were best suited to elicit detailed perspectives held by study participants. However, given the limited sample size and this qualitative approach, our findings are limited in their generalizability to larger physician populations.

An additional limitation stems from our use of hypothetical cases as stimuli for discussion [48]. Despite selecting various attributes in designing our cases, they are not exhaustive of the numerous applications of AI and conditions observable in psychiatric medicine. Although our cases provided a strong anchor for accessible discussion of AI, they also potentially constrained discussion by focusing participants on specific applications and scenarios where these tools may be applied. As a result, our findings may be biased toward certain health conditions such as depression. Furthermore, cases may have introduced confounders that may have influenced physician perspectives. For example, challenges related to the clinical use of pharmacogenomics independent of AI may have biased physician viewpoints [49]. The cases also lacked significant descriptions of the underlying technical approaches (eg, supervised learning and unsupervised learning), technical requirements, or digital architectures related to how information from AI is integrated into electronic health records or other clinical platforms.

### Further Research

Our findings provide several directions for additional research. User research with physicians who are early adopters of psychiatric AI tools might better assess facilitators, barriers, and outcomes related to tool use. Such studies might focus on examining the perspectives of physicians using specific AI tools. In addition, longitudinal research might assess how physician perspectives change over time as AI tools are introduced into clinical practice. These investigations would capture perspective shifts during exposure to AI tools, which is a key limitation of this study. Including varied study methods such as quantitative and mixed methods approaches as well as studying other physician specialties might help characterize differences in practice areas. Sentiment analysis approaches might also more robustly characterize positive and negative attitudes toward the adoption of AI tools. Delphi methods could support identifying the relative importance of certain benefits and risks to physicians as well as consensus for certain design, development, and policy recommendations for psychiatric AI tools. Finally, additional conceptual work might contribute integrative models and frameworks for incorporating AI tools into psychiatric medicine that highlight best practices, ethical considerations, and design standards that address key physician concerns.

### Conclusions

While there is increasing development of AI tools for psychiatric medicine, a comprehensive understanding of physician perspectives lags behind more technical achievements. Identifying and addressing physician concerns will be a key step forward for the design and adoption of AI that is responsive to physician needs. Our study offers a novel look at the benefits and risks physicians see with AI in psychiatric medicine based on clinical case scenarios depicting uses they would likely encounter in their practice. We believe that our participants’ projected challenges and successes for these tools will help better address the needs of physicians as these technologies become further integrated into health care.

## Abbreviations

AI: artificial intelligence
ICD-10: International Statistical Classification of Diseases, Tenth Revision
MDD: major depressive disorder
